# Inflammatory Markers and Sleep Architecture in Sleep Bruxism—A Case-Control Study

**DOI:** 10.3390/jcm13030687

**Published:** 2024-01-25

**Authors:** Michal Fulek, Mieszko Wieckiewicz, Anna Szymanska-Chabowska, Pawel Gac, Rafal Poreba, Iwona Markiewicz-Gorka, Anna Wojakowska, Grzegorz Mazur, Helena Martynowicz

**Affiliations:** 1 Department of Internal Medicine, Occupational Diseases, Hypertension and Clinical Oncology, Wroclaw Medical University, 213 Borowska St., 50-556 Wroclaw, Poland; anna.szymanska-chabowska@umw.edu.pl (A.S.-C.); rafal.poreba@umw.edu.pl (R.P.); anna.wojakowska@umw.edu.pl (A.W.); grzegorz.mazur@umw.edu.pl (G.M.); helena.martynowicz@umw.edu.pl (H.M.); 2 Department of Experimental Dentistry, Wroclaw Medical University, 26 Krakowska St., 50-425 Wroclaw, Poland; m.wieckiewicz@onet.pl; 3 Department of Population Health, Division of Environmental Health and Occupational Medicine, Wroclaw Medical University, Mikulicza-Radeckiego 7, 50-368 Wrocław, Poland; pawel.gac@umw.edu.pl (P.G.); iwona.markiewicz-gorka@umw.edu.pl (I.M.-G.)

**Keywords:** sleep bruxism (SB), inflammation, sleep architecture, inflammatory markers, polysomnography, REM sleep stage

## Abstract

**Background:** Sleep bruxism (SB) is a common sleep-related movement behavior with a multifaceted etiology and a deficiently understood pathophysiology. A recent hypothesis suggests a link between SB and systemic inflammation. The scope of the study was to determine whether bruxers have altered sleep structure and different levels of inflammatory parameters compared to nonbruxers. **Methods:** A total of 83 adults underwent full-night polysomnography. The polysomnograms were evaluated using the American Academy of Sleep Medicine (AASM) guidelines. Then, the blood samples were obtained from the participants by venipuncture and the analyses were performed. The study group was divided based on bruxism episode index (BEI) into two groups: BEI ≤ 4 and BEI > 4. **Results:** In comparison with nonbruxers, the oxygen desaturation index (ODI) was significantly higher in severe bruxers (7.5 ± 11.08 vs. 3.33 ± 5.75, *p* < 0.005), as well as the arousal parameters (7.77 ± 4.68 vs. 4.03 ± 2.97, *p* < 0.001), and the mean oxygen desaturation (3.49 ± 0.69 vs. 3.01 ± 0.67, *p* < 0.05). Moreover, the differences in sleep architecture and deprivation of the deep sleep phase were observed, the non-REM sleep stage 3 was significantly shorter in severe bruxers (*p* < 0.03). Differences were also noted in non-REM sleep stage 1 and REM sleep phase. In the investigated group, there were no statistical differences in inflammatory cytokines levels between bruxers and nonbruxers. **Conclusions:** Sleep bruxism is associated with sleep structure alterations and may be associated with deep sleep phase deprivation. The inflammatory markers are not linearly correlated with the severity of sleep bruxism expressed as BEI.

## 1. Introduction

Sleep bruxism (SB) is one of the cardinal sleep-related movement behaviors [1] with a multifaceted etiology and a deficiently understood pathophysiology [2]. With the prevalence of SB reaching up to 31.4% [3], it becomes one of the major challenges that current sleep medicine is facing. Numerous causes of SB have been reported [4], however despite an infinite endeavor to establish a comprehensive definition of bruxism covering all aspects of this complex topic, the sleep bruxism construct seems to endlessly shift parallel to the extensive growth of knowledge on this subject [5]. It has been converted from a pathology to a motor activity with possibly even physiological or protective relevance [6]. After reaching a consent in 2013 [7], which has then been updated by the international consensus on bruxism in 2018 [8], we seemed to agree SB should no longer be regarded as a parafunctional act of grinding teeth leading to tooth wear or other damage to masticatory system structures [9,10]. It is widely accepted SB is a rhythmic (phasic) or non-rhythmic (tonic) masticatory muscle activity during sleep and it is not a movement disorder or a sleep disorder in otherwise healthy subjects [8].

The RDC/TMD Consortium Network Bruxism Consensus Meeting (“Assessment of Bruxism Status”) on 20 March 2017 has proposed a grading system for categorizing bruxism into three categories: possible, probable, and definite [8]. Basing on a positive interview bruxism is described as “possible”, basing on a clinical examination with or without a positive interview it is “probable”, and basing on a positive instrumental examination with or without a positive interview or positive clinical inspection it is “definite” [8]. In short, SB is regarded not to be a disorder nor disease in healthy individuals [8]. SB can be a harmful as well as a protective behavior, depending on its health outcomes [10]. According to the International Classification of Sleep Disorders, the third edition, bruxism is defined as a “sleep-related movement disorder characterized by teeth grinding or clenching associated with an excessive sleep arousal activity” [11].

The search of bruxism etiology seems to never end [12,13]. There have been proposed theories focusing on the genetic origin [14,15], psychosocial factors [16], and vulnerability to stress or anxiety [17], the role of various neurotransmitters: serotonin [18], dopamine [19], gamma aminobutyric acid (GABA), and noradrenaline [18]. There has been even an observed link between dietary fiber intake and the prevalence of sleep bruxism [20]. Among numerous other potential triggering factors, autonomic nervous system modulation [21] and exposure to exogenous risk factors, e.g., tobacco, alcohol, or drugs, and comorbidities [22] are mentioned. Another risk factor for the increased intensity of SB is habitual coffee consumption [23]. SB can also be associated with simple snoring [24]. Moreover, the prevalence of other sleep-related disorders, like obstructive sleep apnea, sleep-related gastroesophageal reflux disease, periodic limb movement during sleep, restless leg syndrome, REM behavior disorder (RBD) and sleep-related epilepsy increases the incidence of sleep bruxism in comparison with the general population [25]. In a recently performed systematic review, it has been observed sleep bruxism could be associated with systemic inflammation [26]. Taken into account that patients with systemic autoimmune disease are at a substantially higher risk of developing cardiovascular disease (CVD) in comparison with the general population [27], we designed a case-control study to support the foregoing premise.

Foregoing data on the relationship between SB and the systemic inflammation are scarce [26]. Only five original studies have investigated this relationship in a way allowing them to be included in the systematic review [28,29,30,31,32]. Moreover, the literature contains only one study based on polysomnography [32], which is a gold standard in the diagnosis of SB. Due to the heterogeneity of the compared studies, only a qualitative comparison and narrative summary could be performed; however, the results supported the thesis that sleep bruxism could be associated with systemic inflammation [26]. In the last few years, more attention has been paid to the study of sleep architecture in patients experiencing bruxism during sleep, as it has been proven successive micro-arousals increased cardiovascular risk [33]. In the international case-control study of patients presenting with first acute stroke called INTERSTROKE, it has been observed sleep disturbance symptoms were common and they were significantly associated with a graded increased risk of stroke [34]. The proven link between cardiovascular and cerebrovascular health [35,36] and sleep deterioration opened an obvious challenge to investigate the causality between sleep disorders and systemic inflammation, which could be the mediator of the cardio- and cerebrovascular events.

## 2. Objectives

The main hypothesis of the study was bruxers had increased proinflammatory parameters and altered sleep architecture. The scope of the study was to determine whether bruxers have altered sleep structures and different levels of inflammatory parameters compared to nonbruxers.

## 3. Materials and Methods

### 3.1. General Characteristics of the Performed Study

This prospective observational case-control study was conducted in accordance with the amended Declaration of Helsinki. The study was approved by the Ethical Committee of Wroclaw Medical University (no. KB-369/2022). Written informed consent was obtained from all the patients prior to their inclusion in the study. A total of 83 adult Caucasian individuals hospitalized in the Sleep Laboratory of the Clinical Department of Internal Medicine, Occupational Diseases, Hypertension, and Clinical Oncology of Wroclaw Medical University were included in the study. This study has been prepared based on the Strengthening the Reporting of Observational Studies in Epidemiology (STROBE) checklist [37].

Patients with possible bruxism [8] were examined by qualified dentists in the Outpatient Clinic for Temporomandibular Disorders at the Wroclaw Medical University, Poland. Basing on a clinical investigation with or without a positive interview, all the examined individuals were diagnosed with probable SB according to the international consensus on the assessment of bruxism from 2018 [8]. For an assessment of the teeth and oral soft tissue for each individual, a standard physical extra- and intraoral examination was conducted. Among the observed clinical symptoms were abnormal tooth wear and damage to the dental hard tissues (i.e., cracked teeth), hypertrophy of masseter and temporalis muscles, tongue and lip indentations, injury to the inner surface of the cheeks (linea alba), and repetitive damage of restorative work or prosthodontic constructions. The calculator’s standard assumptions for our population were used: population size 3,000,000, confidence level 0.95, fraction size 0.3, and a maximum error of 10%. The required group size was 81. We recruited a group of 83 people, thus the minimum sample size was achieved.

### 3.2. Eligibility Criteria

The inclusion criteria were as followed: age between 18 and 90 years, clinical suspicion of sleep bruxism, and willingness to participate in the study. The following exclusion criteria were applied: unwillingness to provide informed consent or undergo polysomnography; coexistence of respiratory insufficiency, or active inflammation; diagnosis of secondary bruxism associated with neurological conditions; history of treatment with medications potentially interfering with the functions of the nervous and muscular systems; presence of severe mental disorders, cognitive disability, or severe systemic diseases including active malignancy; presence of neurological disorders and/or neuropathic pain; addiction to analgesics and/or drugs that could affect the muscles and breathing; or treatment with foregoing medications.

### 3.3. Polysomnography and Sleep Bruxism Assessment

#### 3.3.1. Polysomnography

All the included patients underwent a single-night full polysomnography (PSG) examination performed with a NoxA1 device (NOX Medical, Reykjavik, Iceland) in the Sleep Laboratory of Wroclaw Medical University. Scoring and analysis of the tests were carried out by a qualified, certified physician, following the AASM guidelines. Polysomnograms were assessed in 30 s epochs in accordance with the 2013 AASM standard criteria for sleep scoring. The PSG outcome variables included sleep latency (SL), total sleep time (TST), sleep efficiency (SE, %), and the percentages of N1 (sleep stage 1), N2 (sleep stage 2), N3 (sleep stage 3), and rapid eye movement (REM) sleep. Abnormal respiratory events were scored based on the pressure airflow signal evaluated in accordance with the standard criteria of the AASM Task Force [38]. The arterial oxygen saturation (SpO_2_) was measured using finger pulse oximetry. Apnea was defined as the absence of airflow for ≥10 s, while hypopnea was defined as a reduction in the amplitude of breathing by ≥30% for ≥10 s with a ≥3% decline in blood oxygen saturation or arousal.

#### 3.3.2. Sleep Bruxism

SB was assessed using bilateral masseter electromyography (EMG) and by audio–video evaluation. Bruxism episode index (BEI) was scored according to the AASM standards [11]. In addition, the three forms of EMG pathways—phasic, tonic, and mixed—were scored. The new SB episodes were scored after at least 3 s of stable EMG and when the activity was at least twice the amplitude of the background EMG [38,39]. The electrodes were placed on the right and left masseter muscles to measure electromyographic activity. EMG bursts within 3 s were considered part of the same episode. Persistent episodes lasting >2 s were rated as tonic bruxism and more than three cyclic EMG increases lasting 0.25–2 s as phase bruxism. Episodes combining the features of a tonic episode and a phase episode were assessed as mixed bruxism. Events that mimicked sleep bruxism in EMG (e.g., coughing, swallowing saliva, or yawning) after video evaluation were excluded from BEI.

### 3.4. Blood Sampling and ELISA

Blood samples were obtained from the participants by venipuncture and the analysis was performed at the Department of Population Health, Division of Environmental Health and Occupational Medicine, Wroclaw Medical University, Poland. The detection and quantification of cytokines were obtained based on the enzyme-linked immunosorbent assay (ELISA). Following the manufacturer’s instructions diligently, we utilized the E0563h, E0079h, E0080h, and E0133h ELISA Kit (EIAab, Wuhan, China) to measure Interleukin-1 (IL-1), Interleukin-6 (IL-6), Interleukin-8 (IL-8), and tumor necrosis factor alpha (TNF-a), respectively. The concentrations of investigated proinflammatory markers were expressed in ng/mL.

### 3.5. Statistical Analysis

The statistical package “Dell Statistica 13.1” (Dell Inc., Round Rock, TX, USA) was used to perform statistical analysis. The arithmetic means and SDs of the estimated parameters were calculated for the quantitative variables. The distribution of variables was examined using the Lilliefors test and the W-Shapiro–Wilk test. For the independent quantitative variables with normal and non-normal distribution, we used the Student’s t-test and the Mann–Whitney U test, respectively. The results were considered to be statistically significant at *p* < 0.05.

## 4. Results

The study group comprised 83 participants. The mean age of participants was 34.48 years. The study group parameters are included in Table 1. The mean BEI of the participants overall was 4.37 ± 3.44. According to the AASM standards [38], we divided participants based on BEI into two groups: BEI ≤ 4—not significant to moderate SB and BEI > 4—severe sleep bruxism. Statistical analysis supported the SB principles: all sleep bruxism parameters: bruxism burst index (BBI), apnea to bruxism, arousal to bruxism, as well as total, phasic, tonic, and mixed bruxism episodes were significantly increased among severe bruxers (Table 2). As some authors split the groups with cut-off of more and less than 2, we have consequently distinguished two subgroups with asymmetric size: BEI < 2 (n = 20) and BEI ≥ 2 (n = 63) and presented the results in Table 3.

Furthermore, we studied the relationship between the differences in BEI and sleep and respiratory parameters in the entire studied group, which showed statistically significant differences. The oxygen desaturation index (ODI) was significantly higher in severe bruxers (7.5 ± 11.08 vs. 3.33 ± 5.75, *p* < 0.005), as well as the arousal parameters (7.77 ± 4.68 vs. 4.03 ± 2.97, *p* < 0.001), and mean desaturation (3.49 ± 0.69 vs. 3.01 ± 0.67, *p* < 0.05). Moreover, the differences in sleep architecture and deprivation of the deep sleep phase were observed, the non-REM sleep stage 3 (N3) was significantly shorter in severe bruxers (*p* < 0.03), differences were also noted in non-REM sleep stage 1 (N1) and rapid eye movement sleep stage (R) (Table 4). There was no statistically significant difference in the concentration of investigated inflammatory markers in groups of patients with BEI ≤ 4/h (n = 49) and >4/h (n = 34) (Table 5). Nor has it been observed in the groups with cut-off point of BEI = 2.

Statistically significant differences were observed in total, phasic, tonic, and mixed bruxism episodes (Table 3).

## 5. Discussion

### Sleep Bruxism as a Behavior

To our best knowledge, this study is the first one to reveal the restriction of deep sleep phase among bruxers. The significant differences in sleep architecture and deprivation of the deep sleep phase have vital consequences [40,41,42]. The N3 sleep stage was significantly shorter in severe bruxers. Fragmented sleep, expressed with a higher arousal index (ArI), was proven to be less efficient in comparison with consolidated sleep [43]. The higher number of arousals is followed by greater sleep phase changes and longer intrasleep wakefulness in sleep bruxers [44]. The increase of SB intensity is linked with increased REM [45]. Our study supports the existing evidence [46] of N1 and REM phase extension in bruxers. Ineffective sleep cannot be regarded only in terms of daytime sleepiness and lack of concentration during the day. The systemic complications involve the development of hypertension and as a result increase overall cardiovascular risk and reduce healthy life expectancy [47,48]. The formidable challenge is aggravated by the fact that symptoms accompanying hypertension such as headaches and tinnitus can equally be the symptoms of SB [49,50].

In recent years, more attention is being paid to the systemic consequences of sleep bruxism. Bruxers have increased blood pressure variability [51], which remained a relevant risk factor for arterial hypertension [52]. The intensity of sleep bruxism is correlated not only with hypertension [51], but it is also a predominant component of altered sleep architecture [53]. Interestingly, the association between oxygen desaturations and bruxism episodes is found only in hypertensives, but not in normotensives [54]. Diagnosis of SB should activate the screening for hypertension to provide early and optimal treatment, preventing long-term cardiovascular events [55]. Sleep fragmentation is another negative appearance observed in patients with SB [56]. Depending on the clinical setting, however, SB can potentially be a harmful as well as a protective behavior [10]. The undisputed association between sleep bruxism and tooth wear [57] is expressed as musculoskeletal pain, fractures, and failures of dental restorations and implants or advanced mechanical tooth wear. However, it needs to be kept in mind that tooth wear is not a pathognomonic sign of active SB [58]. Another already mentioned phenomenon linked with SB was the alteration of sleep architecture [56]. It is expressed as shorter total sleep duration and shorter non-rapid eye movement (NREM) and rapid eye movement (REM) sleep, and higher arousals during REM and NREM sleep [53,59], and what is first made evident by this study—the restriction of N3 sleep stage. Elevated stress and anxiety, which remain the determinants of SB, can modulate sympathetic activity [60]. A growing body of evidence has investigated the association between increased heart rate and arousals which in turn were accompanied by increased sympathetic activity and decreased parasympathetic activity [61]. Patients experiencing tooth clenching during the night are also more likely to suffer from headaches in the morning. The onset of jaw clenching and pain is observed to be often unevenly timed, which emphasizes potential differences in the pathophysiology of those conditions [62]. As emphasized above, SB can also have positive consequences in some patients [63]. Therefore, treatment should be focused on investigating the pathologies, comorbidities, or associated factors that led to the onset of SB instead of treating SB as such [64]. For example, patients suffering from Parkinson’s disease are at higher risk not only of drooling but also of xerostomia. In those patients, as well as in those with gastroesophageal reflux disease (GERD) [65,66], SB can be regarded as a protective behavior, as it enhances the salivation [67]. Noteworthy, there is evidence for a strong link between autoimmune diseases and Parkinson’s Disease (PD) [68,69], which leaves an open door for further research on SB as an immune disorder. Another protective implication of SB is suspending the apnea in patients with obstructive sleep apnea (OSA) [70].

The findings from the recent systematic review [26] based on original studies [28,29,30,31,32] indicated a higher intensity of SB could be associated with higher levels of proinflammatory parameters. Moreover, there have not been found any papers suggesting the inflammatory status in bruxers was comparable to nonbruxers [26]. Recently, there has also been published a systematic review supporting the thesis there was a positive correlation between inflammation and masticatory muscle myalgia [71]. In this context, the results of our study concerning systemic inflammation in bruxers may appear surprising. The reason for such a result may be, on the one hand, an insufficient group of patients enrolled to the study and on the other hand the way the systemic inflammation was measured. Despite definitive evidence on the link between SCI and mortality [72], we seem to lack standardized biomarkers for indicating the presence of health-damaging chronic inflammation [73]. According to the systemic reactivity levels [74], it should be noted in the group with BEI ≤ 4/h as well as in group with BEI > 4/h the mean values of investigated parameters have exceeded what was regarded as a norm [75]. Lack of elevation of the proinflammatory cytokines in patients with severe bruxism can also be a result of the fact that concentrations of SIR factors in blood are usually characterized by non-linearity and non-normal distribution [74].

In our study, we observed a statistically significant difference in mean desaturation between groups with severe SB and moderate SB (3.49% ± 0.69 vs. 3.01% ± 0.67, *p* < 0.05). This could confirm the preliminary report by Dumais I.E. et al. [76] stating the onset of RMMA episodes may be associated with transient hypoxia. According to Suzuki Y. et al. [77], however, the mild and brief oxygen fluctuations occurring prior to RMMA onset may be the reflection of physiological response without significant influence on SB genesis. Whether or not the desaturations are correlated with SB independently of concomitant sleep arousal or body movements needs further investigation.

SCI has the potential of increasing cardiovascular risk [27] and SB, if proven to be an inflammatory-based condition, could also be potentially associated with higher cardiovascular risk. Therefore, the systemic response to sleep bruxism and above all, the causality between sleep bruxism and elevation of proinflammatory markers need further investigation. The disruptive finding arising from our study is the deprivation of deep sleep phase in bruxers. Hopefully, with the portable EMG device [78] and recently published translation guideline [79] for the new tool for bruxism screening—BruxScreen [80], the assessment of sleep bruxism with a high level of accuracy in both awake and sleep bruxism [45] would become more achievable.

Our study is not free from limitations. The most important one is the findings are based only on a single-night polysomnography. The second one was the investigated group of subjects included in the study derived from outpatient clinic for temporomandibular disorders, where no healthy individuals appear. The distinguished groups BEI ≤ 4 and BEI > 4 categorized the participants into subgroups of unequal sizes, n = 49 and n = 34, respectively. Additionally, patients were checked for conditions that may introduce confounding factors. Notably, conditions such as gastroesophageal reflux, obstructive sleep apnea, and TMD, with chronic pain as a potential confounder, have not been accounted for. This could raise challenges in establishing a clear association between sleep bruxism and the reported outcomes. Therefore, the study’s design inherently lacks the capacity to infer causality conclusively. The limitations in the study design undermine the ability to establish direct causal relationships between the variables under investigation. Therefore, caution must be exercised in interpreting the findings within the context of causation.

## 6. Conclusions

1. Sleep bruxism is associated with sleep structure alterations potentially leading to an increase of cardiovascular risk.
2. Sleep bruxism may be associated with deep sleep phase deprivation.
3. The inflammatory markers and/or the sleep architecture perturbances are not linearly correlated with the severity of sleep bruxism, expressed as BEI.
4. The oxygen desaturation index and mean desaturation were significantly higher in severe bruxers.
5. Diagnostic of SB by dentists and preliminary screening of sleep disorders, with posterior reference to a sleep center and specialist in the field, can prevent and protect patient from dangerous health consequences.

## Figures and Tables

**Table 1:** Clinical characteristics of the study group.

	Mean ± SD	95% Confidence Interval
age (years)	34.48 ± 10.7	32.14–36.83
height (m)	1.69 ± 0.09	1.68–1.71
body mass (kg)	66.07 ± 14.53	62.84–69.31
body mass index	22.88 ± 3.97	22.00–23.77
BEI (n/h)	4.37 ± 3.44	3.63–5.12

BEI—Bruxism episode index.

**Table 2:** Sleep bruxism parameters: comparison between the BEI ≤ 4/h and >4/h groups.

	Mean ± SD		
BEI	≤4/h	>4/h	*p* Value
	(n = 49)	(n = 34)	
BEI (n/h)	2.15 ± 1.06	7.64 ± 3.10	*p* < 0.001 *
	(1.85–2.45)	(6.55–8.72)	
BBI (n/h)	2.67 ± 1.70	8.70 ± 6.08	*p* < 0.001 *
	(2.19–3.16)	(6.57–10.82)	
Apnea to Bruxism	0.27 ± 0.38	1.72 ± 2.40	*p* < 0.001 *
	(0.16–0.38)	(0.89–2.56)	
Arousal to Bruxism	0.89 ± 0.80	3.83 ± 2.06	*p* < 0.001 *
	(0.66–1.12)	(3.11–4.55)	
Phasic bruxism episode index (n/h)	0.52 ± 0.54	2.99 ± 2.46	*p* < 0.001 *
	(0.37–0.67)	(2.13–3.85)	
Tonic bruxism episode index (n/h)	1.15 ± 0.64	2.97 ± 1.73	*p* < 0.001 *
	(0.97–1.33)	(2.37–3.57)	
Mixed bruxism episode index (n/h)	0.52 ± 0.43	1.76 ± 1.06	*p* < 0.001 *
	(0.40–0.65)	(1.39–2.13)	

BEI—Bruxism episode index; BBI—bruxism burst index; * *p* statistically significant; Data format: mean ± standard deviation (95% confidence interval).

**Table 3:** Sleep bruxism parameters: comparison between the BEI < 2/h and ≥2/h groups.

	Mean ± SD		
BEI	<2/h	≥2/h	*p* Value
	(n = 20)	(n = 63)	
BEI (n/h)	1.13 ± 0.52	5.45 ± 3.32	*p* < 0.001 *
BBI (n/h)	1.54 ± 1.01	6.30 ± 5.27	*p* < 0.001 *
Apnea to Bruxism	0.28 ± 0.41	1.05 ± 1.91	*p* = 0.069
Arousal to Bruxism	0.67 ± 0.65	2.60 ± 2.09	*p* < 0.001 *
Phasic bruxism episode index (n/h)	0.28 ± 0.49	1.93 ± 2.16	*p* < 0.001 *
Tonic bruxism episode index (n/h)	0.67 ± 0.39	2.29 ± 1.51	*p* < 0.001 *
Mixed bruxism episode index (n/h)	0.27 ± 0.30	1.27 ± 0.98	*p* < 0.001 *

BEI—Bruxism episode index; BBI—bruxism burst index; * *p* statistically significant; Data format: mean ± standard deviation (95% confidence interval).

**Table 4:** Sleep and respiratory parameters in the BEI ≤ 4/h and >4/h groups.

	Mean ± SD		
BEI	≤4/h	>4/h	*p* Value
	(n = 49)	(n = 34)	
AHI (n/h)	3.84 ± 6.86	7.66 ± 12.25	*p* > 0.05
	(1.89–5.79)	(3.38–11.93)	
ODI (n/h)	3.33 ± 5.75	7.53 ± 11.08	*p* < 0.005 *
	(1.69–4.97)	(3.66–11.40)	
Snore (% of TST)	6.66 ± 12.43	9.45 ± 17.84	*p* > 0.05
	(3.13–10.19)	(3.23–15.68)	
TST (min)	415.02 ± 66.60	434.68 ± 41.47	*p* > 0.05
	(396.10–433.95)	(420.21–449.15)	
SL (min)	21.05 ± 23.68	22.34 ± 16.71	*p* > 0.05
	(14.33–27.78)	(16.50–28.17)	
REML (min)	107.28 ± 73.46	101.42 ± 48.67	*p* > 0.05
	(86.41–128.16)	(84.44–118.41)	
WASO (min)	36.85 ± 35.10	31.08 ± 23.85	*p* > 0.05
	(26.88–46.83)	(22.75–39.40)	
SE (%)	85.28 ± 11.89	88.11 ± 6.17	*p* > 0.05
	(81.90–88.66)	(85.95–90.26)	
N1 (% of TST)	4.22 ± 3.48	6.08 ± 4.19	*p* < 0.05 *
	(3.23–5.21)	(4.61–7.54)	
N2 (% of TST)	52.03 ± 8.79	49.87 ± 5.96	*p* > 0.05
	(49.54–54.53)	(47.79–51.95)	
N3 (% of TST)	22.78 ± 6.35	19.74 ± 6.07	*p* < 0.05 *
	(20.98–24.59)	(17.62–21.86)	
R (% of TST)	20.96 ± 5.05	24.32 ± 4.65	*p* < 0.05 *
	(19.52–22.39)	(22.70–25.95)	
ArI (n/h)	4.03 ± 2.97	7.77 ± 4.68	*p* < 0.001 *
	(3.19–4.88)	(6.13–9.40)	
OAI (n/h)	0.22 ± 0.66	1.09 ± 3.84	*p* > 0.05
	(0.03–0.40)	(0.25–2.42)	
MAI (n/h)	0.01 ± 0.06	0.02 ± 0.10	*p* > 0.05
	(0.00–0.03)	(0.01–0.06)	
CAI (n/h)	0.25 ± 0.49	0.27 ± 0.39	*p* > 0.05
	(0.11–0.39)	(0.14–0.41)	
Mean SpO_2_ (%)	94.97 ± 1.33	94.53 ± 2.23	*p* > 0.05
	(94.59–95.34)	(93.76–95.31)	
Min. SpO_2_ (%)	89.88 ± 6.25	87.24 ± 7.60	*p* > 0.05
	(88.11–91.65)	(84.58–89.89)	
SpO_2_ < 90% (%)	0.54 ± 2.70	1.79 ± 3.84	*p* > 0.05
	(0.23–1.30)	(0.45–3.12)	
Mean desat. (%)	3.01 ± 0.67	3.49 ± 0.69	*p* < 0.05 *
	(2.82–3.20)	(3.24–3.74)	
Mean Pulse (bpm)	61.71 ± 8.84	61.61 ± 6.90	*p* > 0.05
	(59.17–64.26)	(59.20–64.02)	

AHI: apnea–hypopnea index, ODI: oxygen desaturation index, TST: total sleep time, SL: sleep latency, REML: REM latency, WASO: wake after sleep onset, SE: sleep efficiency, N1: non-REM sleep stage 1, N2: non-REM sleep stage 2, N3: non-REM sleep stage 3, R: rapid eye movement sleep stage, ArI: arousal index, OA: oral sleep apnea, MA: mixed sleep apnea, CA: central sleep apnea, mean SpO_2_: mean oxygen saturation, Min. SpO_2_: minimal oxygen saturation, Mean desat.: mean desaturation; * p statistically significant. Data format: mean ± standard deviation (95% confidence interval).

**Table 5:** Inflammatory markers; comparison between patients with BEI ≤ 4/h and >4/h.

	Mean ± SD		
BEI	≤4/h	>4/h	*p* Value
	(n = 49)	(n = 34)	
IL-1 (pg/mL)	11.59 ± 2.30	11.33 ± 4.46	*p* > 0.05
	(10.92–12.26)	(9.74–12.91)	
IL-6 (pg/mL)	9.08 ± 1.51	9.37 ± 1.66	*p* > 0.05
	(8.64–9.52)	(8.78–9.96)	
IL-8 (pg/mL)	21.94 ± 13.68	20.35 ± 14.13	*p* > 0.05
	(17.96–25.91)	(15.34–25.36)	
TNF-a (pg/mL)	13.40 ± 6.28	13.16 ± 2.42	*p* > 0.05
	(11.79–16.40)	(12.30–14.02)	

IL-1: Interleukin-1, IL-6: Interleukin-6, IL-8: Interleukin-8, TNF-a: tumor necrosis factor alpha; Data format: mean ± standard deviation (95% confidence interval).

## Data Availability

Data are contained within the article.
